# “The *Cango Lyec* Project - Healing the Elephant”: HIV related vulnerabilities of post-conflict affected populations aged 13–49 years living in three Mid-Northern Uganda districts

**DOI:** 10.1186/s12879-016-2030-0

**Published:** 2016-11-21

**Authors:** Samuel S. Malamba, Herbert Muyinda, Patricia M. Spittal, John P. Ekwaru, Noah Kiwanuka, Martin D. Ogwang, Patrick Odong, Paul K. Kitandwe, Achilles Katamba, Kate Jongbloed, Nelson K. Sewankambo, Eugene Kinyanda, Alden Blair, Martin T. Schechter

**Affiliations:** 1 Uganda Virus Research Institute (UVRI) - HIV Reference Laboratory Program, Entebbe, Uganda; 2 Makerere University, Child Health Development Center, Kampala, Uganda; 3 University of British Columbia, School of Population & Public Health, Vancouver, Canada; 4 School of Public Health, University of Alberta, Alberta, Canada; 5 Uganda Virus Research Institute - International HIV/AIDS Vaccine Initiative (UVRI-IAVI) HIV Vaccine Program, Entebbe, Uganda; 6 St. Mary’s Hospital-Lacor, Gulu, Uganda; 7 Northern Uganda Program on Health Sciences, Kampala, Uganda; 8 Makerere University College of Health Sciences, Kampala, Uganda; 9 MRC/UVRI Uganda Research Unit on AIDS, Entebbe, Uganda; 10 Butabika National Psychiatric Referral Hospital, Nakawa, Uganda; 11 Northern Uganda Program on Health Sciences, c/o Uganda Virus Research Institute, HIV Reference Laboratory, P.O. Box 49, Entebbe, Kampala, Uganda

**Keywords:** HIV, Prevalence, Risk factors, Post conflict, Northern Uganda

## Abstract

**Background:**

The protracted war between the Government of Uganda and the Lord’s Resistance Army in Northern Uganda (1996–2006) resulted in widespread atrocities, destruction of health infrastructure and services, weakening the social and economic fabric of the affected populations, internal displacement and death. Despite grave concerns that increased spread of HIV/AIDS may be devastating to post conflict Northern Uganda, empirical epidemiological data describing the legacy of the war on HIV infection are scarce.

**Methods:**

The ‘*Cango Lyec*’ Project is an open cohort study involving conflict-affected populations living in three districts of Gulu, Nwoya and Amuru in mid-northern Uganda. Between November 2011 and July 2012, 8 study communities randomly selected out of 32, were mapped and house-to-house census conducted to enumerate the entire community population. Consenting participants aged 13–49 years were enrolled and interviewer-administered data were collected on trauma, depression and socio-demographic-behavioural characteristics, in the local Luo language. Venous blood was taken for HIV and syphilis serology. Multivariable logistic regression was used to determine factors associated with HIV prevalence at baseline.

**Results:**

A total of 2954 participants were eligible, of whom 2449 were enrolled. Among 2388 participants with known HIV status, HIV prevalence was 12.2% (95%CI: 10.8-13.8), higher in females (14.6%) than males (8.5%, *p* < 0.001), higher in Gulu (15.2%) than Nwoya (11.6%, *p* < 0.001) and Amuru (7.5%, *p* = 0.006) districts. In this post-conflict period, HIV infection was significantly associated with war trauma experiences (Adj. OR = 2.50; 95%CI: 1.31–4.79), the psychiatric problems of PTSD (Adj. OR = 1.44; 95%CI: 1.06–1.96), Major Depressive Disorder (Adj. OR = 1.89; 95%CI: 1.28–2.80) and suicidal ideation (Adj. OR = 1.87; 95%CI: 1.34–2.61). Other HIV related vulnerabilities included older age, being married, separated, divorced or widowed, residing in an urban district, ulcerative sexually transmitted infections, and staying in a female headed household. There was no evidence in this study to suggest that people with a history of abduction were more likely to be HIV positive.

**Conclusions:**

HIV prevalence in this post conflict-affected population is high and is significantly associated with age, trauma, depression, history of ulcerative STIs, and residing in more urban districts. Evidence-based HIV/STI prevention programs and culturally safe, gender and trauma-informed are urgently needed.

## Background

In Northern Uganda, protracted war between the Government of Uganda and the Lord’s Resistance Army (LRA) resulted in widespread atrocities, rights violations, displacement and death. Between 2004 and 2006, more than 1.8 million people – accounting for over 90% of the population – were forcibly displaced into Internally Displaced People (IDP) camps where they were entirely dependent upon relief aid and services [1]. Signing of the ‘Cessation of Hostilities Agreement’ in 2006 initiated return migration of IDPs to their ancestral homes [2]. Currently, the majority of IDP camps have closed and as of December 2010, more than 90% of IDPs who were encamped at the height of the conflict had returned to their traditional homesteads.

The Uganda AIDS Indicator Survey 2011 estimated an HIV prevalence of 8.3% among people aged 15–49 years in the entire mid-Northern region, nearly double the lowest regional HIV prevalence of 4.1% for mid-Eastern Uganda [3]. Data collected through antenatal care in Gulu district – one of the most conflict-affected areas in Northern Uganda – estimated HIV prevalence to be at 10.3% in 2006 [4, 5]. However, well-established concerns that ANC data may significantly underestimate the true prevalence of HIV remain [6–9]. Further, as available data are limited to the regional level, detailed HIV prevalence estimates in areas and sub-groups most affected by the conflict are urgently required to provide reliable estimates that would guide prevention and care programmatic activities. The new UNAIDS 90-90-90 targets call for rapid scale-up of HIV treatment and an end to the epidemic in 2030. For these goals to be met, substantial improvements in reducing gaps in HIV prevalence data for key populations, including those affected by conflict, are critical.

The relationship between HIV/AIDS and conflict is complex [10]. On one hand, conflict may decrease HIV risk by reducing mobility and improving social service in IDP camp settings [6, 11, 12]. On the other hand, it may increase vulnerability through increased sexual violence, breakdown of social service, and disrupted health infrastructure [12]. War-related vulnerability may make individuals more susceptible to infection (i.e. due to changes in biological and environmental factors), while also increasing the risk of exposure (i.e. population movement, poverty, increased sexual and physical violence) [6]. However, risks and protective factors influencing HIV vulnerability in post-conflict settings are not well understood, particularly the impact of psychological consequences of war and relocation.

Exposure to war-related sexual violence in Northern Uganda has been well documented [13–17], yet the extent to which it has heightened HIV vulnerability in the region is unknown [14]. Estimates suggest that between 25000 and 66000 children aged 6–13 were abducted during the conflict, profoundly impacting the physical and social wellbeing of young people in the post-conflict era [18]. Abductees were forced to serve as child soldiers, labourers, and sex slaves [18]. Premenstrual girls in particular are believed to have been at increased risk of abduction, as they were considered less likely to be living with HIV [19]. Sexual violence has also been documented in and near IDP camp settings, including alarming levels of sexual violence and mass rape by armed forces on the outskirts of camps [13, 16, 20]. Of note, a significant percentage of boys and men were also victims of war-related sexual violence [21–24]. A recent study among young people aged 15–29 living in post-emergency phase transit camps in Gulu district found that the strongest predictor of HIV infection was non-consensual sexual debut, and that this risk factor was reported by over 25% of young women and nearly 8% of young men [25].

With prevailing peace in Northern Uganda, trade between the South Sudan and Northern Uganda is booming. A consequence of this new post-war economy involves cross-border movement of truckers, agricultural traders, and cattle-loaders. Simultaneously, withdrawal of food programs that were central to relief efforts in camps and a recent drought and crop failure raised concerns about high levels of food insecurity reportedly worse than at the height of insurgency [26–30]. There is growing recognition that poverty and food insecurity may be key drivers of the HIV epidemic by increasing sex-related vulnerabilities including subsistence, intergenerational, and coerced sex [6, 31–34]. Further, young people disconnected from traditional livelihoods after prolonged displacement must now find their way in the post-conflict setting. Consequently, increased levels of transactional and subsistence sex have been reported all along the Kampala-Juba highway, including in Gulu, Atiak and Bibia [35]. While sex work was certainly part of life before the war, it is now a source of growing discussion, disquiet, and even tension within some communities due to its relationship to HIV infection.

Concerns of a growing HIV epidemic led Ugandan and Canadian investigators to initiate the *‘Cango Lyec’* Project study involving Acholi people in Northern Uganda at risk of HIV in the aftermath of a long rebel-led civil war. The longitudinal 5-year cohort sought to determine population-wide HIV prevalence and risk factors to inform the development of prevention programs. This paper reports baseline HIV prevalence and correlates of HIV infection from Amuru, Gulu and Nwoya Districts, Northern Uganda.

## Methods

### Study design and setting

This paper reports baseline findings of a 5-year prospective open cohort study involving a representative sample of conflict-affected people living in the former Gulu district, which was later sub-divided to create Amuru, Gulu, and Nwoya Districts, Northern Uganda. Baseline field activities were conducted between November 2011 and July 2012.

### Sample size calculation

The required sample was calculated using the formula: $n={\left(\frac{z}{m}\right)}^2\widehat{p}\left(1-\widehat{p}\right)\times \frac{deff}{R}$ [36]. This gave a total sample size adjusted to 2400 to take care of possible non-response (R) and sampling design effect (*deff*), assuming a design effect (*deff*) of 2 and a response rate (R) of 98.7% based on previous studies that were conducted in rural South West Uganda, ‘*m*’ being the level of precision desired. This sample size would produce a two-sided 95% confidence interval (Alpha = 0.05) with a width ranging from 2.0 to 2.3% around the point estimate when the overall prevalence of HIV infection ranges between 6.4 and 9.1% in all communities combined.

### Selection of study communities and population

A two-stage stratified sampling method was used to randomly select three study communities in each district, one from each settlement category. All communities in the three districts were listed; all major settlements were mapped and categorized as either permanent, transient or displaced. Settlements created to accommodate IDP during the war were categorized as displaced communities; Settlements created to accommodate IDP returning to their overgrown gardens and destroyed houses were categorized as transient communities and settlements which were there before the war and residents were never displaced were categorized to as Permanent. Amuru district had 10 communities (6 permanent, 2 transient, 2 displaced); Gulu district had 16 communities (10 permanent, 2 transient, 3 displaced, 1 pilot community) and Nwoya district had seven communities (4 permanent, 3 transient, 0 displaced). A total of eight study communities were selected from 32 listed communities excluding the pilot community. One community was purposively selected for the purposes of piloting questionnaires and survey tools. Information obtained from residents of this pilot community were not included in the study analyses but was used to identify sensitivities individuals or communities had to some questions and to inform changes to the final survey tools. Communities with large population sizes (>250 adults) like Awach and Layibi were sub-divided by villages\divisions which were randomly selected to represent these communities and contributed household numbers proportional to size of the communities they represent as compared to the populations of other selected communities to fit within the estimated study sample size of 2400 individuals. Only two communities were selected in Nwoya district because it did not have any categorized as ‘displaced’. All households in the selected communities were mapped and a household census was completed to establish the number and the demographic characteristics of all individuals (*N* = 6375). A “take all” approach was used to survey all consenting individuals aged 13–49 years who had been residents in the selected communities for the last month. We used the 13–49 year age-group for two main reasons. 1) To be able to compare our prevalence estimates with the Uganda AIDS Indicator Survey 2011 and 2) to increase our power of estimating HIV incidence since HIV-infections mostly occur in this age range.

### Data collection procedures

The study team was trained on the key components of the study protocol including the study objectives, consenting procedures, administration of the study questionnaire, counselling and the referral process for those respondents found to have HIV, syphilis, PTSD or depression.

### Laboratory testing

Two ELISA tests, Vironostika HIV Uni-Form II plus O (Biomerieux SA, Marcy l’Etoile, France) and Murex HIV-1.2.O (Diasorin S.P.A, Dartford, United Kingdom) were used in parallel to test for HIV infection at the Uganda Virus Research Institute IAVI-laboratory. The Western Blot (Genetic Systems, Bio-Rad Laboratories) was used as a tiebreaker if the ELISA results were discordant. Indeterminate Western Blot results not resolved by the time of this analysis were excluded. Samples screening positive for syphilis using the rapid plasma regain (RPR) test were subjected to a confirmatory *treponema pallidum haemagglutination* test (TPHA).

### Data collection tools

Blood specimen collection was used to determine HIV prevalence and was linked with a questionnaire assessing socio-demographic characteristics, conflict-related experiences and sexual behaviours. Questionnaires were translated into local language (Luo) through a process of forward and backward translation by an experienced team of health professionals working independently. Where there was a wide disparity, translations were discussed and revised to bring out the intended meaning. Given that some questions may elicit memories of significant trauma and victimization, participants who requested care were referred to Trauma Clinics in the study area. Part I and IV of the Harvard Trauma Questionnaire (HTQ) were used to measure post-traumatic stress disorder (PTSD). PTSD scores were calculated using the sum of all answered items in Part IV divided by the number of answered items. Scores ≥2.0 were classified as meeting the criteria for screening positive for PTSD [37–39]. The Hopkins Symptom Check List-25 (HSCL-25) was used to measure major depressive disorder (MDD). Participants with a mean score of ≥1.75 on Part II were classified as meeting the criteria for screening positive for MDD [37–39]. Luo versions of the HTQ and HSCL-25, as developed and validated by Roberts et al. for use in Gulu district, Uganda, were adopted [37, 38]. Calculation of scores on the HTQ and the HSCL was carried out using published guidelines for these instruments. Experiences of war events in Northern Uganda were collected using a 15-item War Trauma Experience Check-list (WETC-15) instrument. Scores on the WTEC-15 were dichotomized; ≤12 vs. >12 severe traumatic events based on a previous study by Roberts [21].

### Statistical analysis

Data were entered in duplicate using Microsoft Access software and analysed using SAS 9.4 software (SAS Institute, Cary, NC, USA). Unadjusted and multivariable logistic regression models were used to determine variables independently associated with HIV infection. All factors including socio-demographic, medical, laboratory, psycho-social and war-related trauma associated with HIV sero-positivity at a ≤0.1 level of significance in the unadjusted models were entered into a multivariable model that adjusted for differences in age, gender, marital status, religion, education, and district of residence. A stepwise regression procedure that dropped, at each step, variables that did not reach the <0.1 level of significance was used to develop the multivariable models excluding factors considered to be on the causal pathway and including factors that were associated with HIV sero-positivity at *p* < 0.05 in the final model for each potential risk factor. A likelihood ratio test was used to check for interactions and determine the model that best fits the data. Unadjusted and adjusted odds ratios of prevalent HIV infection are reported. To account for the additional variance due to the complex two-stage sampling study design that included stratification by the three settlement types and the three study districts, and the unequal selection probabilities and clustering, survey analysis procedures in SAS were used. Communities were the primary sampling units (PSU) and the stratification variables were the settlement type and the district. The sampling weights were based on the selection probabilities at each level of selection and on the proportion of survey participants consenting for interview and HIV testing. For independent categorical variables, we included a missing value category to minimize list-wise deletion of observation in the models. All 61 indeterminate HIV test results (Outcome variable) were considered missing, and were excluded from the analysis.

### Ethics, consent and permissions

Ethical approvals were obtained from the University of British Columbia-Providence Healthcare Research Ethics Board (Canada), Makerere University College of Health Sciences-School of Public Health Science Ethical Committee, Uganda Virus Research Institute-Science and Ethics Committee, and Uganda National Council for Science and Technology. The Office of the President of Uganda issued a letter, which was signed by the Resident District Commissioner in each district and from all participating district authorities. Informed written consent was also obtained from all eligible study participants aged 13–49 years after explaining the objectives and procedures of the study. Parental/legal guardian’s written consent for <18 years with minor’s assent were also obtained. Every participant was properly counselled and consented before being asked to sign or put a fingerprint on the consent form if they were not able to write. Study participants were assured of confidentiality before the start of each interview. All data collected was kept under lock and key only accessible to the research team.

## Results

### Study population

**Fig. 1:**
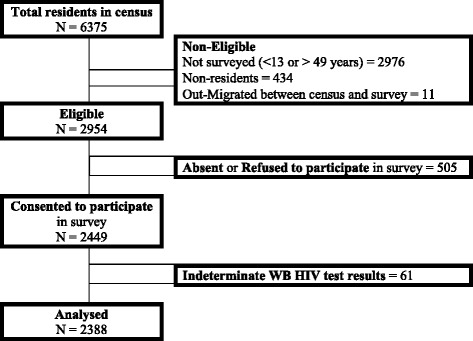
Participant enrolment flow diagram and reason for not enrolling. *Legend:* Study participants’ enrolment flow diagram in the *Cango Lyec* study, mid-northern Uganda districts of Amuru, Gulu and Nwoya, 2011/2012. Approximately 81% of the eligible participants were analysed and 98% of those who consented were analysed

From a household census of 6375 residents, 2976 individuals were not surveyed because they were less than 13 or greater than 49 years old, 434 were non-residents and 11 were absent or had out-migrated between the time of conducting the household census and the survey. A total of 2954 individuals were eligible, of whom 2449 (82.9%) consented to participate (Amuru district = 732, Gulu district = 1159 and Nwoya district = 558). A total of 61 records with indeterminate HIV Western Blot results were excluded from any analysis modelling HIV (Fig. 1).

### Socio-demographic characteristics of the respondents by HIV sero-status

Table 1 shows the distribution of socio-demographic characteristics of all enrolled study participants with determined HIV status. The study population was mostly female (59.7%) and the majority (81.4%) were less than 35 years old with a median age of 25 years (IQR: 18–32). Of the 857 men who responded to the circumcision status question, only 72 (8.4%) self-reported that they were circumcised. Slightly more than two thirds of participants (67.6%) were residing in communities which were formerly displaced or transient camps. Almost one fifth (21.9%) had ever been abducted and only 11.1% had consistently used a condom with their last three sexual partners in the last 12 months. A substantial proportion (71.9%) was Roman Catholic, 53% were currently married, 13.1% had never received any form of formal education, 9.3% reported genital ulcers in the last 12 months and 4.2% (95%CI: 3.3–5.2) tested positive for active syphilis. One in eight participants was staying in a female-headed household and 8.0% were residing in a child (<25 years) headed household.

**Table 1:** HIV-1 sero-positivity by population socio-demographic characteristics and sexual history at baseline

Population characteristics	Total			Weighted (%)	Unadjusted	
	(*N* = 2388)	(%)	95% CI	HIV+ (%)	OR (95%CI)	*p*-value
Age group						
13–19	723	29.1	(23.5–35.5)	15 (2.2%)	1.00	
20–24	443	20.2	(13.1–29.7)	40 (8.5%)	4.24 (1.81,9.93)	0.005
25–29	442	19.6	(16.4–23.4)	58 (14.0%)	7.43 (3.62,15.24)	<.001
30–34	315	12.4	(11.0–14.1)	61 (21.7%)	12.60 (5.98,26.52)	<.001
35+	465	18.6	(14.9–23.1)	103 (23.4%)	13.87 (7.79,24.70)	<.001
Sex						
Male	991	40.3	(34.1–46.8)	77 (8.5%)	1.00	
Female	1397	59.7	(53.2–65.9)	200 (14.6%)	1.84 (1.50,2.25)	<.001
Current marital status						
Never married	739	31.7	(26.6–37.2)	13 (1.8%)	1.00	
Married	1248	53.0	(46.6–59.3)	180 (15.0%)	9.51 (7.51,12.05)	<.001
Separated/divorced	185	8.2	(4.7–14.1)	41 (24.8%)	17.70 (9.90,31.65)	<.001
Widowed	65	2.5	(1.8–3.3)	31 (49.1%)	51.80 (29.11,92.17)	<.001
Missing	151	4.6	(0.4–34.4)	12 (7.4%)	4.28 (1.38,13.28)	0.019
Highest education attained						
Never	350	13.1	(5.2–29.3)	40 (12.6%)	1.00	
Primary	1333	55.5	(47.4–63.2)	182 (14.6%)	1.19 (0.69,2.02)	0.477
Sec/Tertiary/University	638	28.3	(17.7–42.0)	48 (7.7%)	0.58 (0.28,1.18)	0.112
Others	67	3.2	(1.4–7.2)	7 (6.6%)	0.49 (0.05,4.42)	0.468
Religion						
Roman Catholic	1690	71.9	(62.0–80.0)	207 (12.7%)	1.00	
Protestant	358	14.0	(9.4–20.5)	48 (14.1%)	1.13 (0.48,2.66)	0.748
Moslem	28	1.2	(0.5–2.7)	4 (19.6%)	1.67 (0.92,3.04)	0.080
Other	178	9.1	(5.0–15.9)	10 (6.5%)	0.48 (0.21,1.10)	0.073
Missing	134	3.8	(0.2–40.3)	8 (5.9%)	0.43 (0.22,0.87)	0.025
District of residence						
Amuru	703	33.7	(4.7–83.9)	53 (7.5%)	1.00	
Gulu	1139	53.3	(9.0–93.0)	160 (15.2%)	2.21 (1.68,2.91)	<.001
Nwoya	546	12.9	(1.1–66.8)	64 (11.6%)	1.62 (1.49,1.76)	<.001
Community displacement status						
Displaced	333	12.9	(5.5–27.3)	28 (8.6%)	1.00	
Transient	1122	54.7	(20.7–84.8)	148 (14.7%)	1.84 (1.12,3.04)	0.023
Permanent	933	32.4	(8.8–70.6)	101 (9.3%)	1.09 (0.69,1.72)	0.669
Staying in a child (<25) headed household						
No	1658	61.3	(21.7–90.0)	181 (10.7%)	1.00	
Yes	240	8.0	(4.1–15.2)	11 (5.3%)	0.46 (0.30,0.73)	0.005
Missing	490	30.7	(4.8–79.5)	85 (16.8%)	1.69 (1.09,2.61)	0.026
Staying in a female headed household						
No	1524	56.9	(22.2–85.9)	124 (8.0%)	1.00	
Yes	375	12.5	(5.6–25.7)	68 (19.3%)	2.74 (2.21,3.39)	<.001
Missing	489	30.6	(4.8–79.4)	85 (16.9%)	2.32 (1.55,3.48)	0.002
Ever been abducted						
No	1798	78.1	(70.6–84.1)	180 (10.8%)	1.00	
Yes	590	21.9	(15.9–29.4)	97 (17.0%)	1.69 (1.24,2.30)	0.005
Circumcised men (Self report)						
No	785	82.3	(53.6–94.9)	65 (9.1%)	1.00	
Yes	72	8.4	(2.5–25.0)	4 (5.5%)	0.59 (0.11,3.27)	0.487
Missing	134	9.3	(0.5–66.2)	8 (6.0%)	0.63 (0.36,1.13)	0.104
Consistent condom use with last 3 partners in past 12 months						
Inconsistent	1724	71.4	(67.1–75.4)	241 (14.7%)	1.00	
Consistent	231	11.1	(6.3–18.8)	33 (14.1%)	0.95 (0.44,2.03)	0.872
Never had sex	433	17.5	(12.4–24.1)	3 (0.4%)	0.02 (0.00,0.21)	0.005
Genital ulcers in last 12 months						
No	2184	90.7	(88.4–92.5)	212 (10.0%)	1.00	
Yes	204	9.3	(7.5–11.6)	65 (33.0%)	4.44 (3.11,6.34)	<0.001
Active Syphilis						
Negative	2290	95.8	(94.8–96.7)	250 (11.3%)	1.00	
Positive	98	4.2	(3.3–5.2)	27 (32.0%)	3.70 (2.33,5.85)	<0.001
Number of sexual partners ever						
0	441	17.7	(12.7–24.1)	3 (0.4%)	1.0	
1	453	19.5	(15.8–23.9)	19 (4.3%)	12.73 (1.50, 107.85)	0.026
2	424	17.6	(14.1–21.7)	61 (11.7%)	37.80 (5.74, 248.94)	0.003
3+	963	40.8	(30.9–51.4)	193 (21.9%)	79.67 (8.54, 743.23)	0.002
Missing	107	4.4	(1.7–11.0)	7 (6.3%)	19.10 (2.21, 165.23)	0.014

*Legend:* logistic regression data from the *Cango Lyec* study, mid-northern Uganda districts, 2011/2012

NB: % weighted using sampling weights

The prevalence of HIV infection was 12.2% (95%CI: 7.6–18.8) overall; 14.6% (95% CI: 9.3–22.2) in females and 8.5% (95%CI: 5.6–12.7) in males (*p* < 0.001). HIV prevalence was higher in females than that in males in all age-groups below 45 years but this difference was only significantly higher below the age of 30 years (Table 2).

**Table 2:** Prevalence of HIV-1 infection by age and sex *Cango Lyec* study, mid-northern Uganda districts, 2011/2012

Variable	HIV+/Total (Weighted %)	HIV+/Total (Weighted %)	HIV+/Total (Weighted %)	*p*-value
Age	Total	Males	Females	
13–19	15/723 (2.2%)	3/343 (0.8%)	12/380 (3.4%)	0.005
20–24	40/443 (8.5%)	8/185 (4.0%)	32/258 (11.5%)	<0.001
25–29	58/442 (14.0%)	11/161 (9.3%)	47/281 (16.5%)	0.046
30–34	61/315 (21.7%)	15/118 (15.7%)	46/197 (25.0%)	0.152
35–39	46/199 (22.7%)	17/84 (22.6%)	29/115 (22.8%)	0.972
40–44	42/155 (30.1%)	16/63 (27.7%)	26/92 (31.4%)	0.073
45–49	15/111 (14.6%)	7/37 (17.8%)	8/74 (13.0%)	0.655
Total	277/2388 (12.2%)	77/991 (8.5%)	200/1397 (14.6%)	0.0002

*Legend:* HIV prevalence was higher in females compared to males and increased with increasing age with a peak at the 40–44 years in both sexes

NB: % weighted using sampling weights

### HIV infection and mental health

Eight percent (95%CI: 6.4–10.0) of participants had experienced rape or sexual abuse, 14.9% (95%CI: 12.6–17.5) screened positive for MDD, 11.9% (95%CI: 8.8–15.9) screened positive for PTSD, 8.8% reported experience of 12 or more traumatic events ever and 11.7% (95%CI: 9.7–14.0) reported suicidal ideation. HIV sero-positivity was higher among individuals who reported ever experiencing traumatic events like rape or sexual abuse, ill health without medical care, and suicidal ideation within the last 2 weeks. Study participants classified to have MDD (AOR = 1.89; 95%CI: 1.28–2.80), PTSD (AOR = 1.44; 95%CI: 1.06–1.96), war trauma experiences (Adj. OR = 2.50; 95%CI: 1.31–4.79) and suicidal ideation (Adj. OR = 1.87; 95%CI: 1.34–2.61) were more likely to be living with HIV than those classified not to have these conditions (Table 3).

**Table 3:** Association between HIV infection, major depressive disorder, post-traumatic stress disorder, trauma experiences and suicidal ideation

Variable	Total (*N* = 2388)	HIV+ Weighted (%)	Unadjusted OR (95% CI)	^a^Adjusted OR (95%CI)	*p*-value
Major Depressive Disorder *(Mean Depression Scores ≥1.75)*					
No	2027 (85.1%)	193 (10.2%)	1.00	1.00	
Yes	360 (14.9%)	84 (23.4%)	2.70 (1.95, 3.75)	1.89 (1.28, 2.80)	0.006
PTSD *(Mean PTSD Scores ≥2.0)*					
No	2109 (88.1%)	227 (11.2%)	1.00	1.00	
Yes	279 (11.9%)	50 (19.3%)	1.90 (1.30, 2.78)	1.44 (1.06, 1.96)	0.026
12 or more Traumatic events ever					
No	2160 (91.2%)	227 (11.0%)	1,00	1.00	
Yes	228 (8.8%)	50 (23.7%)	2.52 (1.70, 3.73)	2.50 (1.31, 4.79)	0.012
Ever experienced rape or sexual abuse					
No	2189 (92.0%)	226 (11.0%)	1.00	1.00	
Yes	199 (8.0%)	51 (25.6%)	2.80 (1.88, 4.17)	1.80 (0.96, 3.37)	0.063
Ill Health without medical care ever					
No	1639 (69.7%)	162 (10.1%)	1.00	1.00	
Yes	749 (30.3%)	115 (16.9%)	1.82 (1.36, 2.44)	1.59 (1.00, 2.52)	0.048
Suicidal Ideation within last 2 weeks					
Not at all	2093 (88.4%)	218 (11.2%)	1.00	1.00	
Yes - A little/Quite a bit/Extremely	293 (11.7%)	59 (19.2%)	1.88 (1.30, 2.72)	1.87 (1.34, 2.61)	0.003
Missing	2 (0.1%)	0 (0%)		-	-

*Legend:* Unadjusted and after adjusting^a^ for differences in age, sex, marital status, religion, and district of residence, several mental health factors were found to be associated with HIV-infection in three mid-northern Uganda districts population, 2011/2012

### Socio-behavioural, sexual histories and STI related risk factors for HIV

Additional risk factors significantly associated with HIV infection in the multivariable analysis were: older age, residing in an urban district, being married, being separated, divorced or widowed, staying in a female headed household and consistent condom use in the last 12 months (Table 4.) HIV infection was higher among participants who consistently used condoms with their last 3 partners combined in the past 12 months as compared to those who inconstantly used condoms (AOR = 2.10; 95%CI: 1.38–3.18). Consistent condom use in the last relationship was significantly associated with a higher odds of HIV infection (OR = 1.38; 95%CI: 1.22–1.55) but not for the second and third last relationships. HIV sero-positivity among those who were married (AOR = 4.69; (95%CI: 3.25–6.76), separated/divorced (AOR = 9.17; 95%CI: 5.60–15.00) or widowed (AOR = 20.35; (95%CI: 10.27–40.34) considerably exceeds those who were single (never married). Staying in female-headed households was associated with higher HIV sero-positivity (AOR = 2.30; (95%CI: 1.34–3.94). Active syphilis was confirmed among 98 (4.2%) participants. In the multivariable models, genital ulcers in the past 12 months (AOR = 3.33, 95%CI: 2.58, 4.28) and active syphilis (AOR = 3.55; 95%CI: 1.59–7.93) remained significantly associated with HIV (Table 4).

**Table 4:** Unadjusted and multivariable analysis for socio-behavioural, sexual histories and STI related risk factors for HIV infection (*N* = 2388)

Parameter		HIV	Unadjusted		*Adjusted	
	N	Weighted %	OR (95%CI)	*p*-value	OR (95%CI)	*p*-value
Age in years	2388		1.08 (1.06,1.10)	<0.001	1.05 (1.03,1.08)	0.002
Sex						
Female	1397	14.6	1.00		1.00	
Male	991	8.5	0.54 (0.44,0.67)	<0.001	1.17 (0.95,1.45)	0.114
District						
Amuru	703	7.5	1.00		1.00	
Gulu	1139	15.2	2.21 (1.68,2.91)	<0.001	2.20 (1.37,3.54)	0.006
Nwoya	546	11.6	1.62 (1.49,1.76)	<0.001	1.67 (1.52,1.85)	<0.001
Community Displacement status						
Displaced	333	12.9	1.00		1.00	
Transient	1122	54.7	1.84 (1.12,3.04)	0.023	1.68 (0.64,4.36)	0.243
Permanent	933	32.4	1.09 (0.69,1.72)	0.669	1.32 (0.71,2.48)	0.328
Marital status						
Never married	739	1.8	1.00		1.00	
Married	1248	15.0	9.51 (7.51,12.05)	<0.001	4.69 (3.25,6.76)	<0.001
Separated/divorced	185	24.8	17.70 (9.90,31.65)	<0.001	9.17 (5.60,15.00)	<0.001
Widowed	65	49.1	51.80 (29.11,92.17)	<0.001	20.35 (10.27,40.34)	<0.001
Missing	151	7.4	4.28 (1.38,13.28)	0.019	5.74 (0.47,70.37)	0.143
Religion						
Roman Catholic	1690	12.7	1.00		1.00	
Protestant	358	14.1	1.13 (0.48,2.66)	0.748	0.96 (0.46,2.02)	0.904
Moslem	28	19.6	1.67 (0.92,3.04)	0.080	1.99 (0.96,4.12)	0.061
Other	178	6.5	0.48 (0.21,1.10)	0.073	0.35 (0.14,0.86)	0.028
Missing	134	5.9	0.43 (0.22,0.87)	0.025	0.35 (0.04,2.79)	0.268
Staying in a female headed household						
No	1524	8.0	1.00		1.00	
Yes	375	19.3	2.74 (2.21,3.39)	<0.001	2.55 (1.75,3.69)	<0.001
Missing	489	16.9	2.32 (1.55,3.48)	0.002	1.98 (1.23,3.18)	0.011
Staying in a child (<25 years) headed household						
No	1658	10.7	1.00		1.00	
Yes	240	5.3	0.46 (0.30,0.73)	0.005	0.74 (0.40,1.36)	0.274
Missing	490	16.8	1.69 (1.09,2.61)	0.026	-	<0.001
Given/gave money or gifts in exchange for sex in past 12 months						
No	1778	14.9	1.00		1.00	
Yes	21	28.0	2.22 (0.72,6.83)	0.138	2.79 (0.80,9.73)	0.093
Never had sex	411	0.4	0.02 (0.00,0.19)	0.004	-	<.001
Missing	178	5.5	0.33 (0.18,0.62)	0.004	0.38 (0.12,1.19)	0.085
Number of sexual partners in last year						
0	656	6.6	1.00		1.00	
1	1385	13.5	2.21 (1.54,3.19)	0.001	1.19 (0.69,2.04)	0.479
2	217	19.1	3.35 (2.10,5.36)	<0.001	2.37 (1.13,4.98)	0.029
3+	113	15.6	2.63 (1.19,5.82)	0.024	2.14 (0.64,7.13)	0.179
Missing	17	.	-	<0.001	-	<.001
Consistent condom use with the last 3 partners in past 12 months						
Inconsistent	1724	14.7	1.00		1.00	
Consistent	231	14.1	0.95 (0.44,2.03)	0.872	2.10 (1.38,3.18)	0.004
Never had sex	433	0.4	0.02 (0.00,0.21)	0.005	0.21 (0.02,1.85)	0.134
Ever been abducted						
No	1798	10.8	1.00		1.00	
Yes	590	17.0	1.69 (1.24,2.30)	0.005	1.12 (0.70,1.80)	0.578
Genital ulcers in past 12 months						
No	2184	10.0	1.00		1.00	
Yes	204	33.0	4.44 (3.11,6.34)	<0.001	3.33 (2.58,4.28)	<0.001
Active Syphilis						
Negative	2290	11.3	1.00		1.00	
Positive	98	32.0	3.70 (2.33,5.85)	<0.001	3.55 (1.59,7.93)	0.017

*Legend:* Each unit increase in age, district of residence, marital status, residing in a female headed household, condom use, genital ulcers and active syphilis were identified as the main socio-behavioural, sexual histories and STI related factors for HIV infection in this study population

*Adjusted for age, sex, district of residence, religion and marital status

*Note: Sex was forced to remain in the final model

Nested models and the resulting fit characteristics and Likelihood ratio tests

Socio-demographics, sexual histories, STI and HIV (Log Likelihood = −646.4)

Socio-demographics, sexual histories, STI, [sex * age] and HIV (LR Test = 7.13, *p* = 0.076

Socio-demographics, sexual histories, STI, [sex * age * marital status] and HIV (LR Test = 10.63, *p* = 0.06

There was no significant difference in HIV infection between participants who had ever been abducted during the war and those who had never been abducted (*p* = 0.578). Giving or being given money or gifts in exchange for sex in the past 12 months only attained borderline significance (*p* = 0.093); Participants who stayed in a child-headed household (*p* = 0.274); and men who were circumcised were less likely to have HIV but this effect did not attain statistical significance in the multivariable model.

### Sexual experiences at first sexual debut

The median age at first sexual debut among 1955 study participants who reported ever having sex was similar between those with and those without HIV infection (16 years, IQR: 14, 18) but the number of lifetime sex partners was significantly higher in participants with HIV infection (median = 3: IQR: 2–5) than those without HIV infection (Median = 2: IQR: 1–3), *p* < 0.001.

## Discussion

HIV vulnerability among people who have survived the war in Northern Uganda is a critical human rights issue rooted in the length of the war and its atrocities. Our results illustrate that although a majority of the population has returned to their traditional homesteads from IDP camps, Acholi people continue to be severely impacted by a high risk of HIV infection that is significantly associated with war related psychiatric disorder in this post-conflict setting. These findings underscore the urgent need for the implementation of HIV prevention and treatment programs that integrate mental health care and are culturally and gender sensitive.

### HIV prevalence

Findings from this study highlight the enormity of the problem of HIV infection among people residing in Gulu, Nwoya, and Amuru districts of Northern Uganda. HIV prevalence overall was alarmingly high at 12.2%. In contrast, a national AIDS survey conducted in 2011 estimated HIV prevalence in the whole mid Northern region of Uganda to be just over eight percent [40]. Observed estimates of HIV prevalence in ‘*Cango Lyec*’ are markedly higher than the national averages, particularly among women. High prevalence of HIV infection is deeply concerning and demonstrates the continuing crisis of the epidemic in this post-conflict setting.

We observed that district of residence was an important risk factor for HIV infection, with participants residing in Gulu district at least two times as likely to be living with HIV, compared to those in Amuru district. Those living in Nwoya district were also 1.67 times as likely to be living with HIV compared to those in Amuru district. Early in the conflict, the Ugandan government was reluctant to declare Northern Uganda an ‘emergency situation’ and HIV/AIDS funding for the conflict-affected districts remained stagnant. In 2005, the Uganda AIDS Commission and Office of the Prime Minister described the situation in districts impacted by the war as one of poor access, uneven distribution and poorly linked HIV care, treatment, and referral services [41]. With cessation of hostilities, the Government of Uganda announced an official shift in policy towards development-related activities. As a result, many non-government organizations engaged in relief efforts that supported HIV prevention and care closed down operations, leaving significant gaps [42]. Currently, more than 90% of the 2 million internally displaced people in the region have now migrated back to their war-ravaged ancestral villages. Although the conflict is over, district officials remain concerned that inadequate health-care infrastructure and health worker attrition has alarming consequences for HIV prevention as the previously encamped resettle [42]. Our findings clearly indicate the potential for rapid progression of HIV infection in Northern Uganda requiring an aggressive, evidence-based response to HIV prevention that takes into account post-conflict realities.

### Gender

HIV prevalence was much higher among women (14.6%) compared to men (8.5%) in this study (*p* = 0.001). In the multivariable analysis, we observed that young women aged 18 to 34 are at considerably higher risk than young men of the same age, with a 1.64 increase in odds of being HIV positive, after controlling for marital status, district, religion and age. However, the difference was not statistically significant among 35+ year olds. The impact of gender-based disparities in HIV risk were further illustrated by our finding that participants who lived in a female-headed household were more than two and a half times likely to be living with HIV. As conflict-affected young women navigate new environments outside of the bush and IDP camps where they have spent large portions of their lives, district officials are concerned that they face new and undocumented vulnerabilities [25]. With prevailing peace, trade between Northern Uganda and South Sudan has expanded, leading to cross border movement of truckers, agricultural traders, and cattle-loaders into the previously isolated region. These trends have been accompanied by increased transactional and subsistence sex along the Kampala-Juba highway. Young women displaced by war who experienced multiple traumas and never learned agricultural skills may be transitioning into sex work along this new corridor and in rural areas, resulting in the emergence of HIV and STI transmission hotspots. District leaders have raised concerns that families moving away from trading centres back to their ancestral homes leave daughters behind to be closer to schools without adequate socioeconomic support, exposing them to predation. In addition, it has been highlighted that young women have been working in strip/sex clubs and exchanging sex for essential goods, such as sanitary napkins. It is clear from our current work that with the restoration of relative security in Northern Uganda, young women are just as vulnerable to HIV infection, predation, and gender-based violence as they were at the height of the conflict [43]. The drivers of risk have changed, but the vulnerability remains the same.

### War trauma, psychiatric disorder and HIV

This study identified powerful associations between HIV prevalence and war trauma experiences and the psychiatric problems of PTSD, MDD and suicidal ideation among conflict-affected populations. We observed that participants living with HIV were two and a half times as likely to report 12 or more traumatic events. Participants living with HIV were 1.44 times as likely to have probable PTSD, nearly two times as likely to have probable MDD and nearly two times as likely to report suicidal ideation within the last 2 weeks. PTSD may enhance HIV vulnerability by promoting high-risk behaviour, reducing immune function, and interfering with medication adherence [44–46]. However, the relationship between HIV and MDD is complex and bidirectional where persons with MDD are at increased risk for HIV infection, and persons with HIV are at increased risk of developing MDD [47]. Delineating which of these associations is at play in the post-conflict situation of Northern Uganda will be answered by the follow-up component of this study. There is need for more studies in order to understand the link between mental health and HIV vulnerability in the context of war and forced migration [48–50].

Of note, contrary to the speculation that the experience of abduction and its’ associated experience of sexual abuse exacerbated vulnerability to HIV, there is no evidence to suggest that people with a history of abduction are more likely to be HIV positive in this study [48, 51]. However, former abductees in the *‘Cango Lyec’* study were nearly three times more likely to meet the criteria for probable PTSD which in this study has been associated with HIV infection. Anecdotal observations from the region indicate that former abductees experience significant levels of stigma on returning from the bush including the belief that they are HIV infected. Could this stigma and isolation explain the lack of association between abduction and HIV infection? This and many other questions call for further studies in order to understand the complex relationship between war experiences, war related psychiatric disorder and vulnerability to HIV infection. The positive association observed in this study between HIV infection and war related psychiatric disorders calls for the integration of mental health care in HIV prevention and treatment programs in conflict and post-conflict communities.

### Sexually vulnerability

Consistent with existing research, this study observed increased likelihood of HIV among participants with other STIs. It is of great concern that among *‘Cango Lyec’* participants, baseline prevalence of self-reported genital ulcer disease (GUD) in the last year was nearly 10%. Indeed, participants who reported GUD in the past year were more than four times as likely to be living with HIV at baseline. Among participants who tested positive for active syphilis, risk of HIV increased nearly four-fold. Both ulcerative and non-ulcerative STIs have been shown to increase transmission of HIV infection [49, 50, 52, 53]. In a study of monogamous HIV-discordant couples in Rakai, Uganda, risk of HIV transmission was approximately double if one partner had GUD. When both partners had GUD, the risk was nearly four-fold higher [54]. Further, a study of young conflict-affected people aged 15–29 residing in transit camps in Northern Uganda observed that ever having had an STI was associated with a four-fold increase in HIV risk among young women, but was not significantly associated with HIV risk among young men [55]. Active syphilis and other GUD in conjunction with low prevalence of male circumcision is worrisome and may be a warning sign for a rise in new HIV infections in the aftermath of war [56]. These findings highlight the importance of post-conflict HIV prevention interventions that support access to STI diagnosis and treatment.

Compared to those who were single, those who were currently or had ever been married were significantly more likely to be living with HIV in this study. These findings are consistent with other research demonstrating that many new HIV infections in sub-Saharan Africa occur in stable partnerships [57]. However, HIV prevention programs continue to focus on reducing sex and increasing condom use with casual partners.

In addition, participants who reported consistent condom use in the last 3 months were two times as likely to be living with HIV. These findings and the observed extremely low rates (11%) of consistent condom use are concerning, as elsewhere in Uganda [58]. More work must be done to help men and women, including those living with HIV, negotiate condom use with all partners.

### Strengths and limitations

Significant strength of this study includes the ability to determine the HIV and syphilis status using laboratory tests. Efforts were made to enrol all eligible members of the study population. Unfortunately, some individuals were repeatedly absent or refused to participate in the survey. Some of these individuals were more likely to be mobile individuals who have been shown to have higher risks of infection. Reasons for refusing to participate in the survey were not given; some may have refused because of known HIV sero-positive status, we cannot completely rule out selection bias. In addition, the study relies on self-reported behavioural data, there is therefore potential for recall bias, socially desirable reporting, and misclassification of exposure. Responses to historical questions may be influenced by the participant’s ability to recall event(s). Social desirability may lead to an underestimation of some HIV risk behaviours. The effect of memory on these study variables is difficult to assess. Due to the cross-sectional nature of this analysis we are unable to identify cause-effect relationships and temporal sequences. Finally, while the HTQ and HSCL-25 have been demonstrated to be both reliable and valid in this setting, they are screening tools and not diagnostic, which may lead to a conflation in levels of probable PTSD and MDD. On the other hand, ill people might have been more inclined to comply in the expectation of receiving treatment. Despite these limitations, we are confident that due to our recruitment methods and rigorous eligibility criteria our sample is representative of people residing in study communities.

## Conclusion

Evidence of the extreme HIV vulnerability of conflict-affected populations in Northern Uganda observed in this study is an immediate global concern. It is clear from our current work that with the restoration of relative security in Northern Uganda, young women in particular are just as vulnerable to HIV infection, predation, and gender-based violence as they were at the height of the conflict. In addition, war related psychiatric disorders emerge as key risk factors for HIV in this post-conflict community. The drivers of risk have changed, but the vulnerability remains the same. With HIV vaccines unlikely to be available for many years, Acholi people impacted by Northern Uganda’s civil war represent a critical hotspot where specialized interventions are required to prevent transmission and support engagement in HIV care. Meaningful HIV interventions must address the war trauma experiences of this population, the consequent psychiatric disorder and should foster resilience.
